# Global long-term trends and spatial cluster analysis of pancreatic cancer incidence and mortality over a 30-year period using the global burden of disease study 2019 data

**DOI:** 10.1371/journal.pone.0288755

**Published:** 2023-07-20

**Authors:** Maedeh Amini, Mehdi Azizmohammad Looha, Sajjad Rahimi Pordanjani, Hamid Asadzadeh Aghdaei, Mohamad Amin Pourhoseingholi

**Affiliations:** 1 Basic and Molecular Epidemiology of Gastrointestinal Disorders Research Center, Research Institute for Gastroenterology and Liver Diseases, Shahid Beheshti University of Medical Sciences, Tehran, Iran; 2 Social Determinants of Health Research Center, Semnan University of Medical Sciences, Semnan, Iran; 3 Department of Epidemiology and Biostatistics, School of Medicine, Semnan University of Medical Sciences, Semnan, Iran; 4 Gastroenterology and Liver Diseases Research Center, Research Institute for Gastroenterology and Liver Diseases, Shahid Beheshti University of Medical Sciences, Tehran, Iran; Brazilian national cancer institute, BRAZIL

## Abstract

**Introduction:**

Pancreatic cancer (PC) is one of the most fatal malignancies, and its incidence and mortality rates are growing annually throughout the world. In this research, we aimed to investigate the time trends and identify the spatial clusters of incidence and mortality on a global scale over the last 30 years, using the Global Burden of Disease (GBD) study 2019 data.

**Methods:**

Age-standardized incidence and mortality data due to PC were extracted from the GBD study, which was carried out from 1990 to 2019. A Joinpoint regression analysis was utilized to examine trends in the incidence and mortality of PC over the past three decades. As such, spatial analyses were undertaken to detect the spatial distribution and clustering of the metrics globally.

**Results:**

It was observed that both the incidence and mortality rates were higher in males than in females worldwide. The global mortality and incidence rates significantly increased by 0.8% per year over the time of follow-up period (p<0.05). By spatial cluster analysis for mortality, European and North African countries, as well as Greenland were explored as hot spots; while South African and Southeast Asian countries were explored as cold spots. Regarding incidence, hot spots were found in European countries, Southern America, and Greenland; whilst cold spots were determined in Southern Africa and Madagascar.

**Conclusions:**

Collectively, the temporal trends disclosed a gradual rise in PC incidence and mortality rates over the period 1990–2019, reflecting the global health concern. We further found geographical variations in the patterns and identified high- and low-risk areas for incidence and mortality. These findings facilitate the design and implementation of more resource-efficient and geographically targeted treatments. Given the results of the current study, a practical approach to minimizing the future PC burden involves planned population-wide interventions, as well as primary prevention through healthier lifestyles.

## Introduction

Among cancers affecting various organ systems, pancreatic cancer (PC) is a notoriously aggressive tumor type [1]. PC refers to tumors that originate in the cells of the pancreas, a glandular organ located behind the stomach. These tumors proliferate uncontrollably, forming a mass. Despite significant advancements in therapeutic strategies, PC remains one of the most deadly common cancers globally, affecting both females and males [2]. It ranks as the 12^th^ most common malignancy and is categorized as the 7th leading cause of cancer-related deaths, primarily owing to its high fatality rate [3]. As per the GLOBOCAN 2020 report, approximately 495,773 new cases of PC were diagnosed worldwide, accounting for 2.6% of total cancer diagnoses. Of these, 262,865 (5.7%) were males and 232,908 (4.1%) were females. Moreover, in 2020, over 466,000 individuals succumbed to this disease, which accounted for 4.7% of all cancer-related deaths. Among these deaths, 246,840 (5.3%) were males and 219,163 (3.8%) were females [4]. Worldwide, it is projected that the incidence of PC will increase to 18.6 per 100,000 person-years in 2025, with an average annual growth rate of 1.1% [5]. On the other hand, it is expected that PC deaths will become the second leading cause of cancer-related deaths by 2030 [6]. Recent papers have confirmed that the prognosis of PC is extremely poor, with most patients dying 4–6 months after being diagnosed with the disease. However, almost 28% of cases survive for a year, and this percentage falls to about 6% (2%-9%) after 5 years [7]. The principal cause for this may be the fact that PC is generally identified in the late stages, after metastasis, when curative treatment methods are no longer possible [5]. Thereby, PC poses a tremendous global public health problem.

Trends in PC incidence and mortality rates have increased in nearly all countries and territories over the past decades. It is worth noting that the age-standardized rates for both incidence and mortality were greater among males compared with females in all the global burden of disease (GBD) regions during 1990–2017 [8]. On the basis of GLOBOCAN 2020 data, the incidence rate due to PC was higher in developed countries, with the highest incidence and mortality rates observed in Western Europe, and the lowest rates in South Central Asia in 2020. These differences may partly be attributed to the increasing prevalence of lifestyle and environmental risk factors such as obesity, tobacco smoking, diabetes, and alcohol consumption [4, 9]. Furthermore, inherited genetic changes and hereditary factors can play a key role in the risk of developing PC [10]. Given the upward trends of incidence and mortality during different time periods, it is crucial to evaluate the global patterns and temporal trends in PC outcomes. Investigating this issue can provide insights into the long-term effectiveness of previous measures for disease prevention and control over time.

Recent studies from a variety of sources have reported that the incidence and mortality attributed to PC vary greatly across regions and populations [9, 11]. Indeed, it is well evidenced that there are distinct differences in the geographical distribution of PC outcomes. The application of cluster analysis can be a useful approach to indicate these geographic differences. Additionally, it is important to underscore that when the observations are collected from different areas, they may be spatially dependent on each other, exhibiting a sort of spatial autocorrelations that is unexplained within traditional regression approaches. Methodologically, the neglect of spatial autocorrelation may result in biased or under-performing model in health risk assessment [12]. In this respect, numerical approaches have been extensively adopted for spatial cluster detection in health research, which are powerful instruments for exploring clusters of disease events and investigating disease patterns geographically. These approaches estimate the overall degree of spatial autocorrelation in a dataset [13], thereby incorporating the heterogeneity in disease risk for inference. Likewise, mapping PC outcome enables the detection of spatial clusters, providing crucial information on the high burden of disease in specific geographical areas. This information is valuable for designing community-based interventions.

There have been few longitudinal studies conducted to examine the trends of PC outcomes in different world regions. Such studies have been found to be more helpful compared to conventional cross-sectional analyses in assessing temporal patterns. Only one study has focused on the distribution of spatial-temporal clusters in PC mortality, and it was limited to a province in China [14]. A comprehensive understanding of global time trends and spatial cluster analysis of incidence and mortality from PC has not been established yet. Besides, the spatial distribution features vary in different countries and regions because of factors such as geographical location and economic conditions [15]. The absence of elaboration of these subjects in previous works, both in terms of time and space, has led us to take an interest in researching this context. Hence, the remainder of this manuscript is organized as follows. First, we aimed to provide a geographical overview of incidence and mortality, and characterize long-term trends, both overall and by super-region and gender, for the period from 1990 to 2019, based on the GBD study data 2019. The second objective of the present analysis is to examine any significant spatial clustering of PC outcomes during the same time interval at a global level, in order to identify any occurrence of disproportion by location.

## Materials and methods

### Data sources

This was a longitudinal study and the data were related to the annual absolute numbers of incident cases and deaths, and corresponding age-standardized rates of PC which were extracted from the GBD study 2019 online open access database available on the Metrics and Evaluation (IHME) website [16]. IHME provides data via a systematic approach to global, regional, national, and other categories of countries and regions to describe epidemiologic data on various disease, risk factors, and injuries stratified by sex, age, and geographical categories. In total, GBD study 2019 estimated prevalence of exposure and attributable deaths, years of life lost, years of life lived with disability, and disability-adjusted life-years; males, females, and both sexes combined; and 204 countries and territories that were grouped into 21 regions and seven super-regions. Cancer estimation in GBD 2019 utilized 929 193 cancer, location, and year-specific sources of data, of which 767 514 (82.6%) were from vital registration systems, 155 542 (16.7%) from cancer registries, and 6137 (0.7%) from verbal autopsy reports. The GBD 2019 study used a Cause of Death Ensemble model (CODEm) approach that combined data from vital registration systems, cancer registries, and verbal autopsy reports to estimate mortality across several submodels. For each cancer, sex-specific CODEm models generated mortality estimates across locations, years, and age groups. These cancer mortality estimates were then scaled to align with the total mortality for all causes of death, which was separately estimated in GBD 2019. The GBD cancer incidence was estimated by taking mortality estimates from the second step described previously and dividing by MIR estimates from the first step described previously for each cancer type, sex, location, year, and 5-year age group [17]. GBD 2019 study is in agreement with the Guidelines for Accurate and Transparent Health Estimates Reporting (GATHER) statement and relies on various data sources for each disease. After that, data is processed and revised to correct biases and modeled to generate the estimates [18]. A detailed description of the PC incidence and mortality estimates for the GBD are already published [8]. For all estimates in GBD, 95% uncertainty intervals (UIs) were reported. Uncertainty was propagated via each step of the cancer estimation process, with UIs indicating the 2.5th and 97.5th percentiles of the distribution of 1000 draws at each step. More information on general GBD methods can be found in the GBD 2019 summary papers [19, 20]. The waiver of informed consent was reviewed and approved by the University of Washington Institutional Review Board [17]. Therefore, no approval from an ethics committee or informed consent from patients is required for this study.

### Statistical analysis

Primarily, for the purpose of describing and assessing the age-standardized incidence rate (ASIR) and age-standardized mortality rate (ASMR) due to pancreatic cancer, the estimated annual percentage change (EAPC) was adopted in our literature. EAPC can quantify the trends in ASIR and ASMR and was stratified by sex from 1990 to 2019. This is a summative and broadly used measure of the trends during a specified time interval. A linear regression line was fitted to the natural logarithm of the rates with calendar year as an independent variable that is, *y* = *α*+*βx*+*ε*, where *x* referred to calendar year, and *y* = ln(rate). Then, EAPC can be computed as 100×(exp(*β*)−1), and its 95% confidence interval (CI) also calculated based on the empirical quantile method. If the EAPC value and the lower boundary of the 95% CI were both higher than 0, the age-standardized rates were regarded as growing trend over the selected years. By contrast, both the EAPC value and the upper boundary were lower than 0, suggesting a downward trend over time. Otherwise, the trends were deemed to be stable [21]. This statistic was implemented by R software (version 4.1.2, R core team). Accordingly, trends in overall ASIR and ASMR were evaluated using joinpoint regression analysis. This is a non-linear form of analysis to identify trends in the disease burden of PC that allows for the determination of time points. For fitting this model, we applied the Joinpoint regression software version 4.7.0.0 which was provided by the United States National Cancer Institute Surveillance Research program. This software tracks trends in data over the study period and fits the simplest model possible by linking multiple line segments on a logarithm scale. These segments were referred to “joinpoints” with the simplest model (i.e., 0 joinpoints) being a straight line. As more joinpoints were added, each of them was tested for significance through a Monte Carlo permutation approach. For each joinpoint, an annual percentage change (APC) was estimated along with 95% CI. The average annual percent change (AAPC) was also calculated as the weighted average of APCs to provide a summary measure of the trend for the whole time period [22, 23]. In all analyses, we assigned the significance level as α = 0.05, with p < 0.05 regarded statistically significant.

In the next step of data analysis, the spatial pattern and cluster identification was performed for all world countries separately for men, women, and both genders. Spatial cluster analysis plays an important role in visualizing and quantifying geographical variation in patterns of disease burden. The Global Moran’s *I* index was utilized to measure the correlation among neighboring observations, to detect the patterns and the levels of spatial clustering among neighboring districts. Values of this statistic range from -1 to +1. If it was zero value, the incidence/mortality distributed randomly around the world. The more away *I* is from 0, the stronger (positive or negative) the spatial autocorrelation. When *I* > 0, the disease distribution is positive for spatial autocorrelation and vice versa. A positive autocorrelation implies that values in one area are similar to in neighboring areas, while a negative autocorrelation declares that, if one area has a high incidence/mortality rate, the neighborhood areas have low incidence/mortality rate. The Global Moran’s *I* is is computed by $I=\frac{N}{S_{O}}\sum_{i}\sum_{j}\omega_{ij}\frac{\left(x_{i}-u)(x_{j}-u\right)}{\sum_{i}{\left(x_{i}-u\right)}^{2}};\;S_{O}=\sum_{i}\sum_{j}\omega_{ij}$, in which *N* is the number of districts; *ω*_*ij*_ is the element in the spatial weight matrix corresponding to the observation pair *i*, *j*; and *x*_*i*_ and *x*_*j*_ are the observations for areas *i* and *j* with mean *u* [13].

The spatial analytical technique also can be used to identify the location of spatial anomalies (hot spots and cold spots). Hot spot and cold spot refer to the occurrence of the high and low burden of disease that clustered together on the map, respectively [24]. The optimized hot spot analysis (OHSA) is an accurate, valid, and novel tool for finding the spots in different countries of world. This index is based on the Getis-Ord Gi* that automatically chooses the optimal distance band value in which clustering intensity is maximized according to the incremental spatial autocorrelation. Hence, OHSA is the best likely outputs for discerning hot spots and cold spots, which can be revealed at 90%, 95%, and 99% CI. The Getis-Ord Gi* calculates a Z-score where a significant positive Z-score (Gi*) shows the accumulative of high values and the formation of a high-risk cluster, whereas a significant negative Z-score represents the accumulation of low values and the formation of a low-risk cluster. Importantly, if the Z-score is near zero, spatial clustering is not existent [25, 26]. Notably, the mapping, dividing geographical areas into different zones, and creating cut-points for the legends on maps were based on the natural breaks (Jenks) (or Jenks optimization) approach. Furthermore, the level of data aggregation was based on the world countries. It is a data classification method designed to establish the best arrangement of values into multiple clusters [27]. ArcGIS v.3.16.3 (ESRI, Redlands, CA, USA) was employed for spatial distribution analyses (https://spatial.uchicago.edu/geoda).

## Results

### Rates and time trends in incidence and mortality due to PC across the globe and super-regions

The global ASIR indicated a slightly significant incrementing trend in this period, with an overall EAPC of 0.83 (95% CI: 0.78–0.87) for both in males and females. As such, the estimates of ASMR increased from 5.34 (95% UI: 5.07–5.52) per 100,000 person-years to 6.62 (95% UI: 6.11–7.46) per 100,000 person-years over this period, with an EAPC of 0.77 (95% CI: 0.73–0.81). In terms of sex, males experienced a greater ASIR compared with females in 1990 and 2019. Specifically, the global ASIR in males was approximately 1.33 times greater than in females in 2019. Meanwhile, the EAPC of ASIR was higher among males than females (0.84 (95% CI: 0.79–0.90) vs. 0.81 (95% CI: 0.77–0.85), respectively) throughout the whole study period. Furthermore, males were more likely to suffer from PC than females around the world. Accordingly, among all PC deaths in the world, males accounted for 52.37% and females accounted for 47.62%, resulting in a male-to-female ratio of 1.33 in 2019. In males, PC led to a 1.69% (95% UI: 1.62–1.74%) increase in deaths in 2019 compared to 1990, with the ASMR increasing from 6.12*10^5^ (95% UI: 5.81–6.42) in 1990 to 7.55*10^5^ (95% UI: 7.00–8.79) in 2019 (EAPC = 0.79; 95% CI: 0.74–0.85). A significant upward trend was also detected in the ASMR of PC in females (EAPC = 0.74; 95% CI: 0.71–0.77), and deaths grown by nearly 1.67-fold (95% UI: 1.53–1.79%) during the same period (Tables 1 and 2).

**Table 1:** The incident cases and age-standardized incidence rates from pancreatic cancer, as well as their temporal trends by sex and super-region from 1990 to 2019.

		1990		2019		1990–2019
		Incident cases No. (95% UI)	ASIR per 100,000 (95% UI)	Incident cases No. (95% UI)	ASIR per 100,000 (95% UI)	EAPC of ASIR (95% CI)
World						
	Total	197347.99 (188604.03, 203971.11)	5.22 (4.96, 5.46)	530296.75 (486175.15, 573635.16)	6.57 (6.00, 7.09)	0.83(0.78,0.87)
	Female	93295.72 (88533.66, 96901.56)	4.52 (4.26, 4.79)	250393.36 (223820.17, 275352.20)	5.71 (5.11, 6.28)	0.81(0.77,0.85)
	Male	104052.26 (99197.91, 108990.84)	5.99 (5.71, 6.26)	279903.39 (256008.81, 303427.20)	7.49 (6.85, 8.12)	0.84(0.79,0.90)
CEEECA						
	Total	32305.61 (31076.57, 33659.81)	6.79 (6.52, 7.78)	53494.69 (49215.57, 57659.31)	8.51 (7.83, 9.16)	0.65(0.53,0.81)
	Female	15239.61 (14368.71, 16421.21)	5.14 (4.85, 5.54)	25387.32 (22860.28, 28017.14)	6.59 (5.93, 7.29)	0.78(0.65,0.92)
	Male	17066.00 (16616.03, 17968.09)	9.20 (8.95, 9.69)	28107.36 (25556.04, 30675.99)	11.04 (10.04, 12.56)	0.49(0.32,0.65)
HI						
	Total	105277.71 (100849.78, 107499.88)	8.72 (8.36, 8.91)	220848.67 (196750.32, 242213.24)	10.19 (9.10, 11.15)	0.61(0.55,0.65)
	Female	52781.19 (49648.93, 54381.48)	7.33 (6.94, 7.54)	111017.56 (94842.84, 124128.50)	8.92 (7.80 9.94)	0.74(0.71,0.79)
	Male	52496.51 (51122.75, 53345.66)	10.44 (10.13, 16.61)	109831.11 (98735.02, 121552.80)	11.56 (10.39, 12.84)	0.42(0.37,0.46)
LAC						
	Total	8816.52 (8498.10, 8816.52)	4.33 (4.13, 4.45)	32775.38 (29512.75, 35907.93)	5.90 (5.34, 6.44)	1.07(0.96,1.18)
	Female	4402.31 (4208.34, 4537.44)	4.21 (3.97, 4.36)	17004.29 (15115.93, 18857.29)	5.68 (5.06, 6.26)	1.01(0.88,1.15)
	Male	4414.20 (4285.43, 4527.37)	4.43 (4.26, 4.56)	15771.09 (14142.01, 17403.21)	6.12 (5.51, 6.75)	1.13(1.05,1.22)
NAME						
	Total	4518.57 (3840.51, 5331.35)	2.70 (2.27, 3.19)	22236.50 (19272.98, 25716.35)	5.33 (4.64, 6.12)	2.52(2.33,2.67)
	Female	1691.06 (1450.80, 2090.37)	2.09 (1.77, 2.63)	8778.43 (7371.550, 10180.62)	4.41 (3.69, 5.19)	2.65(2.57,2.74)
	Male	2827.51 (2265.03, 3589.42)	3.29 (2.64, 4.15)	13458.07 (11660.79, 15594.46)	6.20 (5.43, 7.16)	2.39(2.16,2.62)
SA						
	Total	7581.95 (6135.46, 8935.49)	1.42 (1.14, 1.68)	38730.75 (34042.52, 43731.61)	2.86 (2.51, 3.24)	2.45(2.35,2.56)
	Female	2895.59 (2316.70, 3644.37)	1.16 (0.90, 1.48)	19123.29 (15850.60, 22411.77)	2.78 (2.32, 3.27)	3.06(2.92,3.21)
	Male	4686.35 (3530.27, 5999.11)	1.66 (1.24, 2.11)	19607.46 (16233.85, 23082.42)	2.94 (2.44, 3.47)	2.02(1.95,2.09)
SAEAO						
	Total	33929.83 (30223.69, 37663.31)	2.99 (2.67, 3.37)	145113.31 (126581.05, 165495.71)	5.46 (4.76, 6.26)	2.27(2.12,2.42)
	Female	14185.27 (12343.33, 16080.88)	2.45 (2.13, 2.76)	60461.51 (50268.47, 71697.83)	4.32 (3.59, 5.12)	2.01(1.86,2.14)
	Male	19744.55 (16503.77, 23161.31)	3.60 (3.04, 4.18)	84651.80 (69347.53, 101702.30)	6.76 (5.58, 8.46)	2.50(2.33,2.66)
SSA						
	Total	4917.78 (4196.56, 5576.02)	2.43 (2.08, 2.77)	17097.42 (15181.29, 19333.42)	3.95 (3.56, 4.41)	1.60(1.53,1.66)
	Female	2100.67 (1787.90, 2490.40)	2.07 (1.76, 2.47)	8620.92 (7450.33, 9847.70)	3.80 (3.30, 4.39)	2.06(2.02,2.11)
	Male	2817.11 (2311.86, 3283.03)	2.79 (2.30, 3.24)	8476.49 (7363.74, 9738.55)	4.11 (3.60, 4.68)	1.22(1.12,1.33)

Note: *UI* Uncertainty Interval; *ASIR* Age-standardized incidence rate; *EAPC* Estimated Annual Percentage Change; *CI* Confidence Interval; *CEEECA* Central Europe, Eastern Europe, and Central Asia, *HI* High Income, *LAC* Latin America and Caribbean, *NAME* North Africa and Middle East, *SA* South Asia, *SAEAO* Southeast Asia, East Asia, and Oceania, *SSA* Sub-Saharan Africa

**Table 2:** The death cases and age-standardized mortality rates from pancreatic cancer, as well as their temporal trends by sex and super-region from 1990 to 2019.

		1990		2019		1990–2019
		Death No. (95% UI)	ASMR per 100,000 (95% UI)	Death No. (95% UI)	ASMR per 100,000 (95% UI)	EAPC of ASMR (95% CI)
World						
	Total	198050.91 (189328.90, 204762.56)	5.34 (5.07, 5.52)	531107.11 (491948.19, 566536.85)	6.62 (6.11, 7.46)	0.77(0.73,0.81)
	Female	94739.12 (89322.34, 98183.67)	4.64 (4.34, 4.82)	252933.60 (225846.18, 273819.66)	5.77 (5.15, 6.24)	0.74(0.71,0.77)
	Male	103311.78 (98381.20, 108763.78)	6.12 (5.81, 6.42)	278173.51 (257504.91, 298745.01)	7.55 (7.00, 8.79)	0.79(0.74,0.85)
CEEECA						
	Total	32988.70 (31723.08, 34386.86)	7.00 (6.71, 7.29)	55073.43 (50226.10, 59699.95)	8.74 (7.97, 9.46)	0.64(0.51,0.79)
	Female	15977.37 (15051.38, 17212.27)	5.38 (5.06, 5.81)	26809.41 (24070.14, 29521.51)	6.88 (6.20, 7.57)	0.77(0.64,0.91)
	Male	17011.33 (16566.34, 17902.98)	9.41 (9.13, 9.93)	28264.01 (25413.28, 30995.32)	11.24 (10.12, 12.29)	0.48(0.32,0.64)
HI						
	Total	104195.34 (99468.66, 106572.21)	8.61 (8.21, 8.84)	213898.08 (193069.88, 225910.34)	9.69 (8.90, 16.18)	0.46(0.43,0.49)
	Female	52715.88 (49243.05, 54405.18)	7.24 (6.81, 7.46)	107722.22 (93859.81, 115766.87)	8.42 (7.54, 8.97)	0.57(0.54,0.62)
	Male	51479.45 (50107.83, 52332.27)	10.32 (9.99, 17.51)	106175.85 (99851.43, 110648.63)	11.09 (10.46, 11.54)	0.30(0.27,0.34)
LAC						
	Total	9087.30 (8727.73, 9322.18)	4.09 (3.92, 4.21)	33958.87 (30743.64, 37037.14)	5.65 (5.08, 6.19)	1.03(0.92,1.13)
	Female	4585.69 (4361.41, 4735.44)	3.95 (3.74, 4.48)	17823.66 (15894.83, 19649.58)	5.40 (4.80 5.99)	0.96(0.83,1.09)
	Male	4501.61 (4359.66, 4625.94)	4.23 (4.08, 4.35)	16135.20 (14534.29, 17860.30)	5.90 (5.29, 6.51)	1.10(1.01,1.18)
NAME						
	Total	4593.98 (3889.31, 5424.33)	2.84 (2.37, 3.36)	22277.14 (19357.43, 25691.38)	5.49 (4.78, 6.31)	2.42(2.25,2.59)
	Female	1740.74 (1484.94, 2172.28)	2.22 (1.87, 2.83)	8869.50 (7453.34, 10280.70)	4.58 (3.84, 5.28)	2.56(2.47,2.65)
	Male	2853.23 (2294.88, 3614.75)	3.44 (2.75, 4.33)	13407.63 (11668.48, 15494.71)	6.37 (5.59, 7.33)	2.32(2.09,2.54)
SA						
	Total	7736.11 (6242.63, 9124.88)	1.52 (1.22, 1.81)	40012.01 (35017.29, 45582.35)	3.04 (2.65, 3.45)	2.41(2.31,2.51)
	Female	2963.57(2378.78, 3791.73)	1.25 (0.98, 1.62)	19881.72 (16392.34, 23534.45)	2.97 (2.43, 3.49)	3.01(2.88,3.15)
	Male	4772.53 (3597.34, 6102.64)	1.78 (1.31, 2.25)	20130.29 (16818.32, 23654.65)	3.11 (2.60, 3.65)	1.98(1.92,2.05)
SAEAO						
	Total	34369.12 (30550.47, 38437.22)	3.15 (2.81, 3.48)	148207.45 (129127.01, 169171.48)	5.68 (4.95, 6.47)	2.22(2.07,2.37)
	Female	14569.83 (12558.02,16560.49)	2.59 (2.25, 2.94)	62831.26 (52595.34, 74606.18)	4.55 (3.81, 5.38)	1.97(1.83,2.12)
	Male	19799.29 (16680.57, 23304.30)	3.79 (3.25, 4.42)	85376.19 (70004.31, 102876.84)	7.00 (5.80, 8.37)	2.43(2.27,2.59)
SSA						
	Total	5080.32 (4372.88, 5745.31)	2.61 (2.24, 2.97)	17680.10 (15687.82, 19936.26)	4.25 (3.80, 4.74)	1.61(1.55,1.67)
	Female	2186.01 (1874.44, 2590.67)	2.24 (1.90, 2.68)	8995.79 (7838.70, 10286.30)	4.11 (3.60, 4.64)	2.08(2.04,2.12)
	Male	2894.31 (2386.34, 3383.68)	2.99 (2.46, 3.48)	8684.31 (7541.33, 9891.73)	4.41 (3.86, 4.97)	1.22(1.12,1.33)

Note: *UI* Uncertainty Interval; *ASMR* Age-standardized mortality rate; *EAPC* Estimated Annual Percentage Change; *CI* Confidence Interval; *CEEECA* Central Europe, Eastern Europe, and Central Asia, *HI* High Income, *LAC* Latin America and Caribbean, *NAME* North Africa and Middle East, *SA* South Asia, *SAEAO* Southeast Asia, East Asia, and Oceania, *SSA* Sub-Saharan Africa

**Fig 1:**
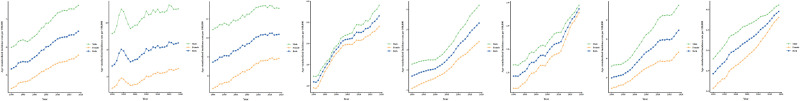
Time trends for pancreatic cancer, in terms of age-standardized incidence rates per 100,000 person-years globally and in seven super-regions during 1990–2019, by gender. **(A)** Global. **(B)** Central Europe, Eastern Europe, and Central Asia. **(C)** High Income, **(D)** Latin America and Caribbean. **(E)** North Africa and Middle East. **(F)** South Asia. **(G)** Southeast Asia, East Asia, and Oceania. **(H)** Sub-Saharan Africa.

**Fig 2:**
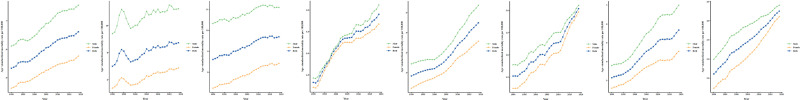
Time trends for pancreatic cancer, in terms of age-standardized mortality rates per 100,000 person-years globally and in seven super-regions during 1990–2019, by sex. **(A)** Global. **(B)** Central Europe, Eastern Europe, and Central Asia. **(C)** High Income, **(D)** Latin America and Caribbean. **(E)** North Africa and Middle East. **(F)** South Asia. **(G)** Southeast Asia, East Asia, and Oceania. **(H)** Sub-Saharan Africa.

For different geographic areas, High-income (HI) had the highest number of PC incidence cases and the highest ASIR (10.19 per 100,000, 95% UI: 9.10–11.15) in 2019 for both genders. During the past three decades, PC incidence cases incremented in all super-regions, with the most significant increasing trends observed in North Africa and Middle East (NAME) (97.4%, 95% UI: 91.8–104.4%) with an EAPC of 2.52 (95% CI: 2.33–2.67). PC deaths mostly occurred among HI countries in 2019. Besides, the PC outcome in terms of mortality was highest in this region (9.69 per 100,000, 95% UI: 8.90–16.18) in 2019. Indeed, this region ranked as the first high-risk area for PC. Notably, the growth speed of ASMR was faster in NAME (EAPC = 2.42, 95% CI: 2.25–2.59) and South Asia (SA) (EAPC = 2.41, 95% CI: 2.31–2.51) compared to the remaining regions throughout the observation period. In the female population, ASIR and ASMR displayed the greatest increasing trends in SA with EAPCs of 3.06 (95% CI: 2.92–3.21) and 3.01 (95% CI: 2.88–3.15), respectively from 1990 to 2019. Subsequently, Southeast Asia, East Asia, and Oceania (SAEAO) was the super-region that experienced the most rapid rise in ASIR (EAPC = 2.50; 95% CI: 2.33–2.66) and ASMR (EAPC = 2.43; 95% CI: 2.27–2.59) considering the entire analyzed period among males (Tables 1 and 2). As shown in Figs 1 and 2, ASIRs and ASMRs related to PC had ascending trends in all GBD super-regions, and the pattern of trends generally resembled between sexes over the same time period.

**Fig 3:**
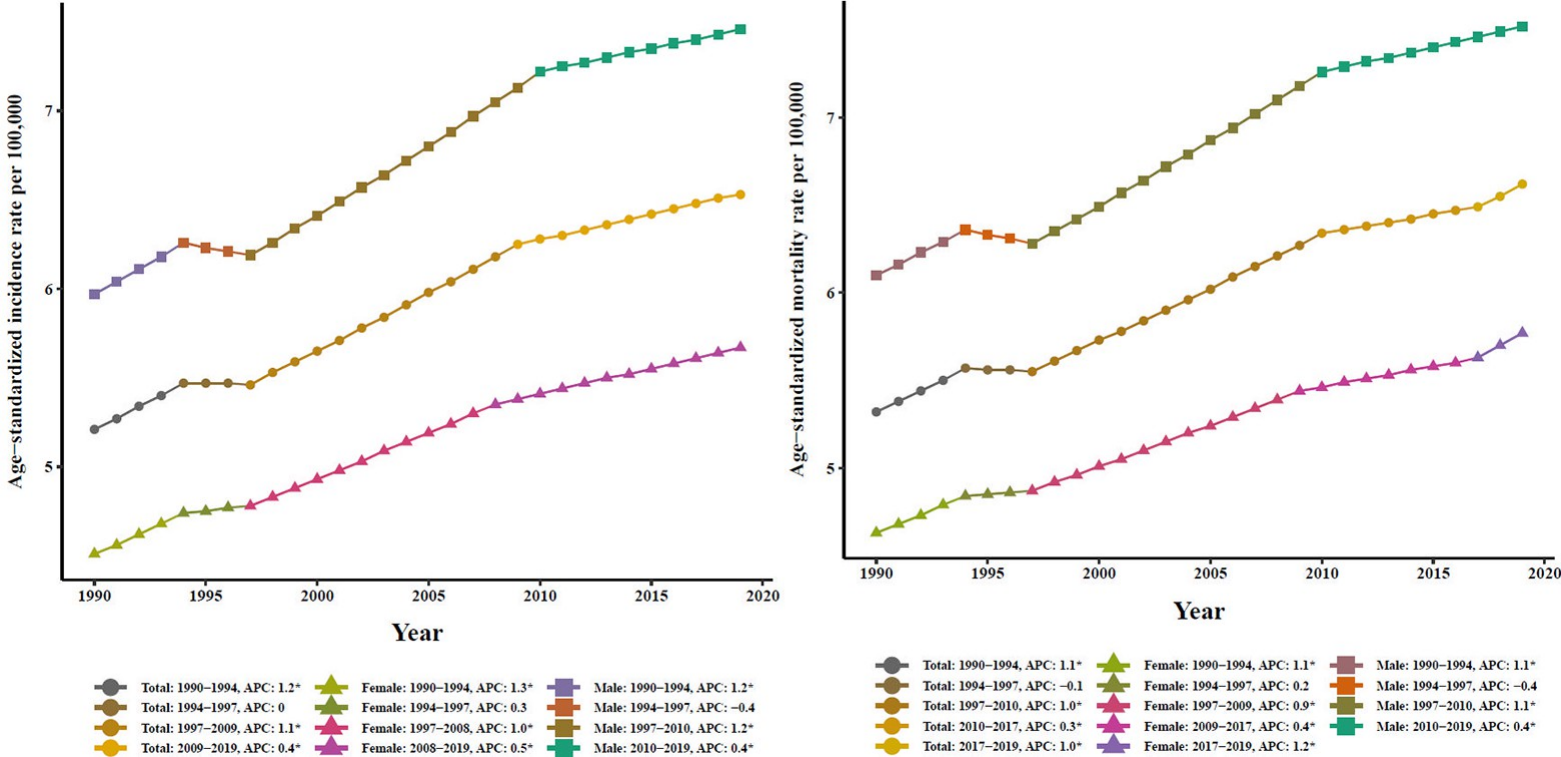
Trend analysis results of joinpoint regression models for pancreatic cancer age-standardized **(A)** incidence and **(B)** mortality rates in males, females, and both sexes worldwide from 1990 to 2019.

The method used recognized joinpoints according to the model with up to five change points. Initially, joinpoint analysis did not reveal significant cut-points during 1994–1997, indicating stabilization of the curves for ASIRs and ASMRs (p>0.05). However, there were significant increases in overall AAPC values for ASIR [0.80% (95% CI: 0.70–0.90%)] and [0.80% (95% CI: 0.60–0.90%)] and ASMR [0.80% (95% CI: 0.60–0.90%)] and [0.70% (95% CI: 0.60–0.80%)] from PC in the world were over the past 30 years, which were similar in females and males. Nonetheless, among females and males, these temporal patterns have varied during particular periods. Specifically, among males, there were significant increases in ASIRs from 1990 to 1994, 1997 to 2010, and 2010 to 2019, with yearly increments of 1.20% (APC = 1.20, 95% CI: 0.80–1.50%), 1.20% (APC = 1.20, 95% CI: 1.10–1.30%), and 0.40% per year (APC = 0.40, 95% CI: 0.30–0.50%), respectively (Table 3). Importantly, the ASIR and ASMR exhibited more pronounced growth (larger APC) from 1990 to 1994 and from 2017 to 2019, respectively compared to other time intervals for females and males (Fig 3).

**Table 3:** Joinpoint trend analysis of age-standardized incidence and mortality rates due to pancreatic cancer worldwide, stratified by gender, during 1990–2019.

Metric	Trend	Female		Male		Both	
		Year	APC (95%CI)	Year	APC (95%CI)	Year	APC (95%CI)
Incidence							
	Trend 1	1990–1994	1.30*(0.90, 1.60)	1990–1994	1.20*(0.80, 1.50)	1990–1994	1.20*(0.90, 1.60)
	Trend 2	1994–1997	0.30(-0.90, 1.50)	1994–1997	-0.40(-1.50, 0.80)	1994–1997	0(-1.10, 1.10)
	Trend 3	1997–2008	1.0*(0.90, 1.10)	1997–2010	1.20*(1.10, 1.30)	1997–2009	1.10*(1.00, 1.20)
	Trend 4	2008–2019	0.5*(0.40, 0.60)	2010–2019	0.40*(0.30, 0.50)	2009–2019	0.40*(0.38, 0.50)
	**AAPC**	**1990–2019**	0.80*(0.70, 0.90)	**1990–2019**	0.80*(0.60, 0.90)	**1990–2019**	0.80*(0.70, 0.90)
Mortality							
	Trend 1	1990–1994	1.10*(0.80, 1.40)	1990–1994	1.10*(0.70, 1.40)	1990–1994	1.10*(0.80, 1.40)
	Trend 2	1994–1997	0.20(-0.60, 1.10)	1994–1997	-0.40(-1.50, 0.70)	1994–1997	-0.10(-1.00, 0.90)
	Trend 3	1997–2009	0.90*(0.89, 10)	1997–2010	1.10*(1.07, 1.20)	1997–2010	1.0*(0.98, 1.10)
	Trend 4	2009–2017	0.40*(0.30, 0.50)	2010–2019	0.4*(0.30, 0.50)	2010–2017	0.30*(0.20, 0.50)
	Trend 5	2017–2019	1.20*(0.30, 2.10)			2017–2019	1.0*(0, 1.90)
	**AAPC**	**1990–2019**	0.80*(0.60, 0.90)	**1990–2019**	0.70*(0.60, 0.80)	**1990–2019**	0.80*(0.60, 0.90)

Note: *APC* Annual Percentage Change; *AAPC* Average Annual Percent Change; *CI* Confidence Interval. *Significantly different from 0 at alpha = 0.05 (p < 0.05). There are 1+(number of trend) joinpoints for each model.

### Global spatial analysis

**Fig 4:**
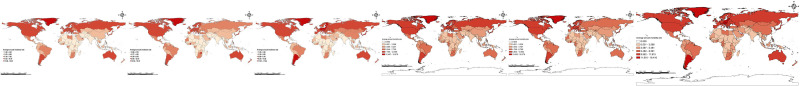
Spatial distribution of the average annual age-standardized incidence and mortality rates due to pancreatic cancer, stratified by sex in the world, 1990–2019. **(A)** Average annual incidence rate in Total. **(B)** Average annual incidence rate among females. **(C)** Average annual incidence rate among males. **(D)** Average annual mortality rate in total. **(E)** Average annual mortality rate among females. **(F)** Average annual mortality rate among males.

Fig 4 presents the geographical distribution of the average annual ASIRs and ASMRs due to PC during a total period of 30 years (1990–2019), stratified by gender. In the analyzed period, the average annual ASIR and ASMR were greater in countries located in the northern hemisphere (such as North American and European countries) than in southern hemisphere countries (such as African and Asian countries). On average, Australia had an estimated ASIR of 5.41 per 100,000 and an ASMR of 5.55 per 100,000, which were higher compared to the globe rates. The countries with the highest average annual ASIRs in both males and females included Greenland (19.01), Monaco (16.48), United Arab Emirates (13.85), Uruguay (11.97), and Czech Republic (11.89). In terms of mortality, Greenland (19.57), Monaco (16.33), United Arab Emirates (14.52), Uruguay (12.46), and Czech Republic (11.95) also had the highest average annual ASMRs. By contrast, Ethiopia (1.20), Guinea (1.47), Bangladesh (1.58), Yemen (1.62), and Somalia (1.63) reported the lowest average annual ASIRs in both genders. Similarly, the lowest average annual ASMRs occurred in the same countries but with different estimates: Ethiopia (1.30), Guinea (1.55), Bangladesh (1.69), Yemen (1.71), and Somalia (1.74). Moreover, the highest average annual ASIRs were found in Greenland (17.94), Monaco (14.86), United Arab Emirates (14.46), Czech Republic (14.42), and Hungary (14.36) among males. For females, the rates were highest in Greenland (19.73), Monaco (17.56), Palau (16.65), United Arab Estimates (12.50), and Uruguay (10.42). On the other hand, the lowest average annual ASIRs were recorded in Guinea (1.08), Ethiopia (1.48), Haiti (1.74), Nepal (1.81), and Bangladesh (1.85) for males, and in Ethiopia (0.88), followed by Somalia (1.01), New Guinea (1.15), Yemen (1.22), and Bangladesh (1.25) for females. In total, the average mean annual ASIR was higher in males than in females (6.23 vs. 4.47). Furthermore, the greatest average annual ASMRs for males were mainly identified in Greenland (18.40), the United Arab Emirates (15.14), Monaco (14.72), the Czech Republic (14.48), and Hungary (14.47). However, the lowest rates were found in Guinea (1.16), Ethiopia (1.61), Haiti (1.85), Nepal (1.97), and Bangladesh (1.98). Among women, the highest average annual ASMRs were observed in Greenland (20.37), Monaco (17.38), Palau (17.37), the United Arab Emirates (13.23), and Uruguay (10.95). In contrast, the lowest average annual ASMRs were identified in Ethiopia (0.94), Somalia (1.08), New Guinea (1.22), Yemen (1.29), and Bangladesh (1.35).

**Fig 5:**
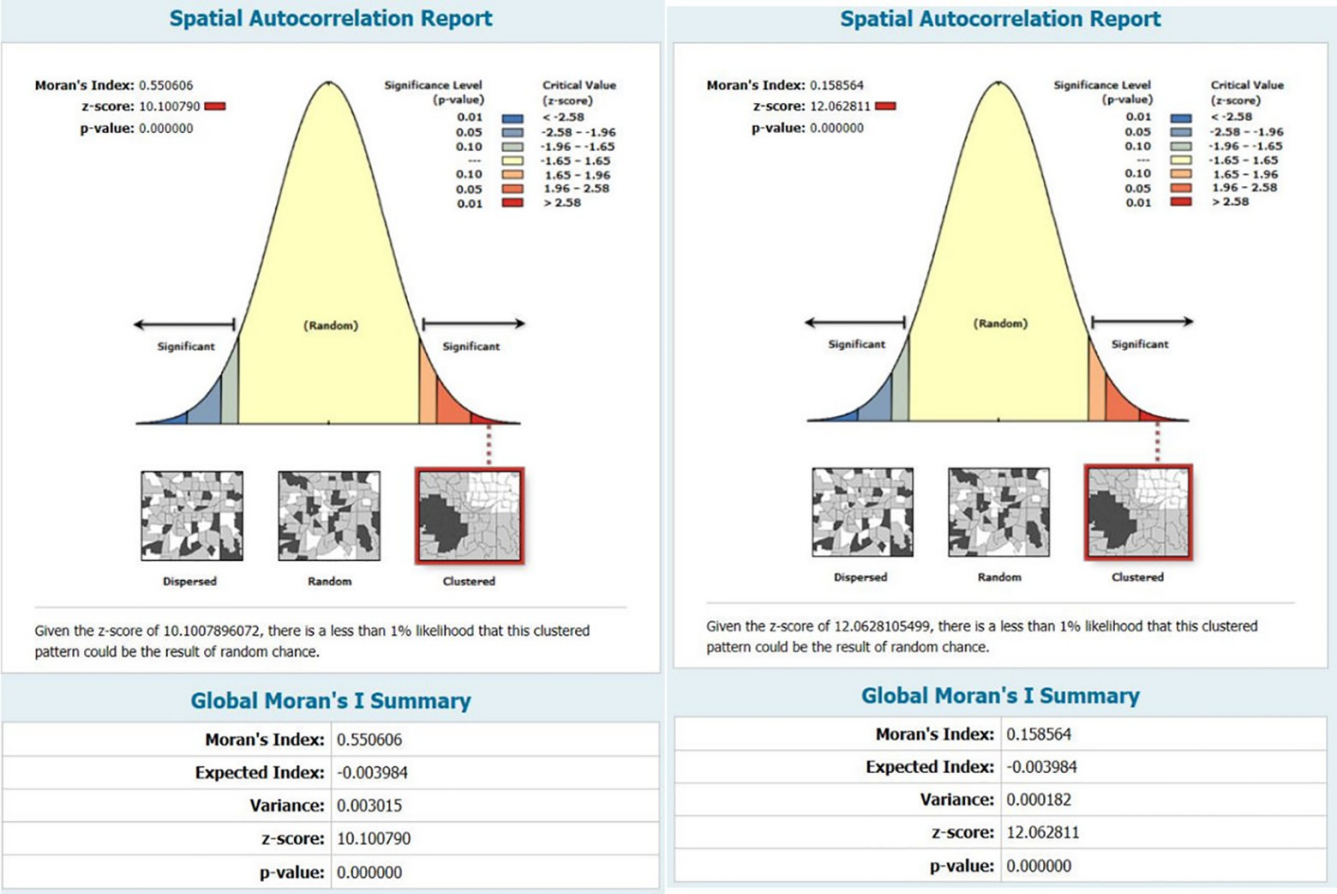
Global spatial autocorrelation of pancreatic cancer age-standardized **(A)** incidence and **(B)** mortality based on the Global Moran’s I Index.

**Fig 6:**
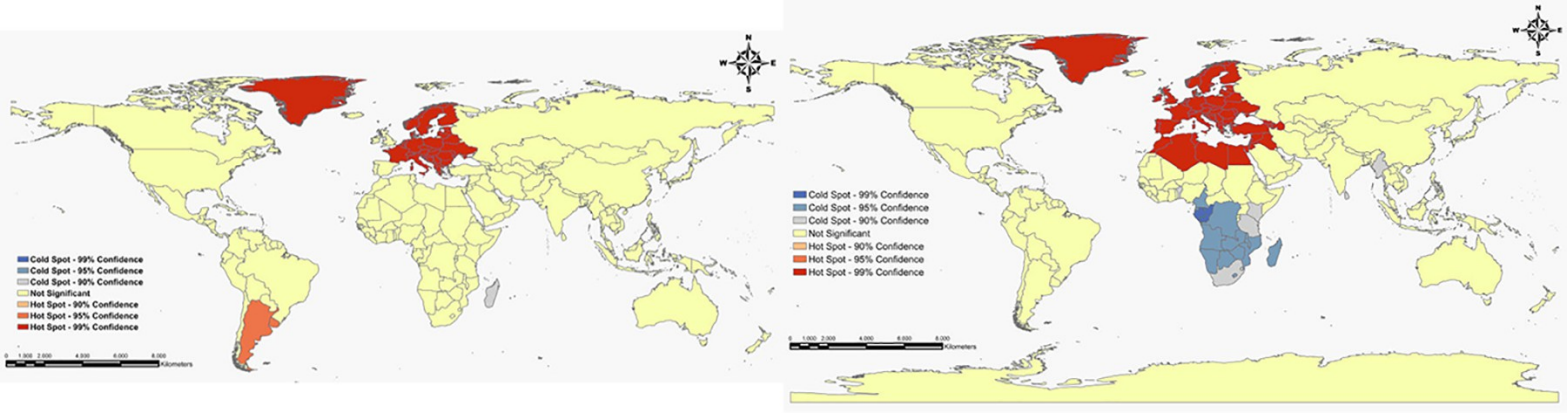
Hot spots (clusters of high-risk) and cold spots (clusters of low-risk) of pancreatic cancer incidence and mortality by sex in the world, 1990–2019. **(A)** Hot spots and cold spots of incidence rate in Total. **(B)** Hot spots and cold spots of mortality rate in total.

All univariate spatial autocorrelations of Global Moran’s I indexes were positive and statistically significant in terms of both ASIR (I = 0.55, p<0.001) and ASMR (I = 0.55, p<0.001), suggesting a severity of spatial dependence among the ASIRs and ASMRs with similar patterns and the rates. Likewise, these results indicate a high clustering tendency of incidence and mortality rates in the world, as opposed to randomly or regularly spaced distribution (Fig 5). Subsequently, the OHSA statistic was used to identify hot spots and cold spots of PC ASIR and ASMR. The optimal distance band for ASIR and ASMR based on this statistic, was estimated to be 1246 km and 1050 km, respectively. The hot spots and cold spots of the rates, with various CIs (90%, 95%, and 99%), are visualized in Fig 6. At first glance, the countries in hot spots of ASIR were primarily located in Europe, South America, and Greenland, while the countries in the cold spots were mainly situated in South African and Madagascar. Additionally, the hot spots of ASMR were observed in countries located in European, North African, and Greenland, whereas countries of South African and Southeast Asian were regarded as cold spots. It should be noted that all hot spots and cold spots were determined in northern hemisphere and southern hemisphere, respectively. Besides, the spatial patterns of hot spots and cold spots of ASIR and ASMR in males and females were exactly the same for both sexes.

## Discussion

As regards the incidence and mortality rates in the past decades, PC poses a significant challenge for the future around the world, despite the progress made in declining the burden of many other cancers. In the current work, when considering different continents worldwide, it is widely acknowledged that the PC ASIR and ASMR exhibit substantial variation among regions. This finding in stark consistent with accumulating evidence that has identified such regional disparities. According to a report, North America and Eastern Europe presented rates of 6.9 and 6.8 per 100,000 people, respectively, while South Africa and Central Asia had rates below 1 death per 100,000 people in 2016 [28]. Although the etiology of PC has been extensively studied, there is no clear clarification for the differences in the rates of incidence and mortality across various regions of the world. However, the geographic inequalities in PC outcomes can be attributed to multiple reasons, such as exposure to lifestyle and environmental risk factors, insufficient healthcare resources, and changes in the utilization of different types of diagnostic modalities [29]. A more recent study has shown that tobacco smoking may contribute to these variations, whilst others suggest dietary habits and obesity as possible factors [30]. Additionally, differences in the incidence rates may be influenced by the quality of cancer registry data, as completeness and precision vary by region and country.

Looking at the ASIR and ASMR of PC in 2019, it was confirmed that the highest ASIR and ASMR tended to be predominate in HI, while the rates were the lowest in countries of SA. One possible explanation for the higher rates is the greater proportions of aging population, unhealthy lifestyle habits, lower rates of solar radiation, and metabolic disorders. Similarly, according to the GLOBOCAN 2018, the highest incidence rates of PC were found in North American and Western European countries, whereas the lowest ASIR was reported in Africa [31]. A previous GBD study in 2017 found that the ASIRs were highest in Greenland and Uruguay as High-income countries, and the lowest were detected in Bangladesh and Sao Tome and Principe [8]. In another study, Carioli et al. revealed that PC remained the most common cause of neoplastic deaths with high rates in the European countries over the last two decades, affecting both men and women [32]. Surprisingly, despite recent advances in diagnosis and treatment, primarily through echography and magnetic resonance imaging in High-income countries, previous literature suggested that this had little impact on PC mortality [33]. Nevertheless, further research is needed to support these findings in this context.

Gender-wise, males outstripped females in terms of ASI and ASM rates across all regions in the past decade. It should be acknowledged that various reports have consistently concluded that males have higher rates compared with females in different parts of the world. In a past report written by Bray et al., the global ASIRs and ASMRs were 5.5 and 5.1 per 100,000, respectively, among males, and 4.0 and 3.8 per 100,000, respectively, among females in 2018 [31]. Alghamdi et al. in their study demonstrated that the overall ASIR for males was higher than that for females [1]. A study conducted in China observed ASI and ASM rates of 52.2 per 100,000 and 45.6 per 100,000, respectively, for men, and 37.9 per 100,000 and 33.8 per 100,000, respectively, for women in 2015 [5]. Moreover, available mortality data from the GBD study 2017 suggested that approximately 51.9% of PC deaths occurred among men, while 48.1% occurred among women [8]. Although the exact reasons for the differences in incidence and mortality rates related to PC between genders are largely unclear, there are hypotheses to explain such differences. Some authors support the idea that environmental or occupational risk factors (e.g., polycyclic aromatic hydrocarbons, nickel compounds, and pesticide), as well as lifestyle factors, may be source of these differences [34, 35]. Large studies have declared smoking as the main environmental risk factor for PC, with its prevalence in men being five times greater than that in women worldwide [8, 9]. In addition, risk factors such as type 2 diabetes, obesity, and alcohol consumption might be attributed to the different PC incidence and mortality rates in males and females. Even so, much more work is needed to determine the role of genetic and other important contributing factors in explaining the observed differences in PC outcomes between the sexes.

Our study also presented regional time trends of PC incidence and mortality. As verified herein, upward trends were seen for ASIR and ASMR in all super-regions. It was noted that the sharpest and slowest significant increases in ASIR and ASMR were detected in countries of NAME, with averages of 2.52% and 2.42%, respectively, and in HI with averages of 0.61% and 0.46%, respectively throughout the study period for both genders. Since the etiology of this neoplasm is complex and not well understood, it is challenging to find out the causes of the rise in the incidence and mortality of PC in these countries. However, a number of epidemiological studies have believed that the prevalence of diabetes mellitus, rapid growth in the rate of obesity, and high prevalence of tobacco use play important roles in the increasing trend of PC incidence in countries of NAME such as United Arab Emirates, Oman, Iran, Iraq, and Lebanon [1]. Coincident with the conclusion of our research, the incidence and mortality rates due to PC have exhibited an ascending trend in various countries and regions over the last few decades. This can be partially explained by improvements in diagnostic procedures, the proliferation of unhealthy lifestyles, the aging population, and the subsequent more frequent identification of silent tumors at an early stage of development.

A previous study suggested a weak association between Helicobacter pylori infection and PC mortality, although the extent of this effect remains unclear [34]. In Saudi Arabia, it was detected that the ASIR of PC increased with age between 2004 and 2015 [1]. In a study covering the geographic trends in the incidence and mortality, Are et al. observed similar patterns based on the GLOBOCAN data 2012. They found statistically significant rises in the burden of PC for the Western Pacific region, and European countries accounted for the highest incidence and mortality over 15-years [36]. Dramatically, the substantial increasing patterns in these countries may indicate a growing prevalence of risk factors associated with globalization, urbanization, economic development, and the increasing incidence of pancreatic cystic neoplasm [3]. As such, it is noteworthy that living standards, health care services, and medical knowledge have improved in most countries in these regions, and the average life expectancy has exceeded 80 years. This issue might result in an increase in PC outcomes, especially in older age groups [37]. Pourshams et al. documented that high-income Asia Pacific and Central Europe had smaller increases in age-standardized rates of incidence and deaths from 1990 to 2017 compared with many other regions. Moreover, the Caribbean Andean Latin America, and Central Asia had the highest percentage change in both incidence and mortality rates in this 27-year period [8].

A retrospective study evidenced a large burden of pancreatic malignancies in East Asia and Pacific, as well as in Europe and Central Asia regions. Surprisingly, the mortality rates of PC in the East Asia and Pacific regions were 20-fold higher compared to the Middle East and North Africa [38]. Findings from another study manifested that, among continents of the world, Africa (114.1%), followed by Latin America and Caribbean (99.3%), had the highest incidence of PC, while the lowest incidence was registered in Europe (29.3%). The same trend was observed for mortality rate, with the highest and lowest rates being recorded in Africa and Latin America and the Caribbean and Europe, respectively. Several reasons contribute to this significant increase in incidence and mortality in these countries. For example, in undeveloped countries like Africa, socioeconomic factors may have a significant impact on the trends due to limited access to improved diagnostic tools and therapies [30]. As such, Japan, as a high-income country, has been falling behind some countries in control measures for PC. Based on the existing report in this country, the trends in the mortality rate for both males and females have incremented during 1980–2016 [39]. Thus, more efforts should be spent to promote preventive measures for known risk factors.

In joinpoint analysis stratified by sex, the ASIRs and ASMRs gradually grew on average by 0.80% and 0.70%, respectively, for males, and 0.80% and 0.80%, respectively, for females in the period 1990–2019 globally. Accordingly, the ASI and ASM patterns for 7 super-regions reported similar significant upward trends annually throughout the period comprehended between 1990 and 2019. The growing trends are undoubtedly the most alarming matter in our research and disclose a bleak and concerning epidemiological scenario for PC in the world and continents [40]. On the other hand, although the explanation for this similarity in the trends is not clear, it may be attributed to changes in the exposure to risk factors shared by both genders. Our results also illustrated that among women, the most marked statistically significant increases were observed in South Asia with 3.06% and 3.01% respectively, for incidence and mortality. In men, the largest positive trends of incidence and mortality have taken place in SAEAO by 2.50% and 2.43%, respectively, during the last three decades. These observations declare that the rises in PC outcome trends were prominent among women from most super-regions (except LAC and SAEAO), albeit the majority of cases and rates occurred among men. Some published articles concluded that the detected steeper mortality increase at the regional level for women could be due to greater prevalence of obesity and a more marked increment in the metabolic syndrome with population aging linked to menopause and sex hormones [10, 37]. Meanwhile, there are multiple pathogenic mechanisms regarding the role of weight gain in increasing the risk of PC. For example, obesity might be accompanied by physical inactivity, an unhealthy diet, and an unhealthy lifestyle [41]. Worldwide, the prevalence of obesity is growing at an alarming rate and is predicted to reach 18% among males and surpass 21% among females. This data shows that it is essential for policymakers to promote the maintenance of a healthy body weight [42]. Regarding the current obesity pandemic in various regions of the world, it is important to emphasize that obesity is considered a strong promotor of diabetes mellitus. Several epidemiological studies have suggested that the ever-increasing prevalence of diabetes, especially in higher-income countries, may play an important role in the ascending trend of PC ASIR in males and females [43, 44]. Besides, there is evidence confirming that the type of alcohol consumption can affect the PC risk in both genders [45]. Some other studies have verified the contribution of cigarette smoking to PC, which partly explains the increasing trends among men and women. It has been reported that the prevalence of daily smoking across the world were 25% and 5.40% for men and women, respectively [46].

The spatial distributions of the greatest average annual incidence and mortality rates of PC were predominantly concentrated in countries with better socioeconomic status, such as North American and European countries. It was further shown that the low-income countries, e.g., African, Asian, and Middle Eastern countries, had the lowest average annual incidence and mortality rates. These findings also observed in a study conducted by Rawla et al. at the global level. They explored more than half of deaths were focused in the most developed countries [30]. Although the causes of the slight geographic different distributions in mean PC incidence and mortality are insufficiently known, it may be attributed to environmental factors and/or the exposure to certain risk factors associated with socioeconomic status. Numerous published articles have concluded that tobacco smoking, dietary style, obesity, and the change in the use of various diagnostic tools between developed and undeveloped geographic areas may have a significant impact on these discrepancies [47, 48]. With respect to the results of the spatial analysis, there was a high spatial autocorrelation and clustering tendency of incidence and mortality in the world. Specifically, we noted that high-risk clusters of PC outcomes mainly occurred among countries located in the northern hemisphere, whilst low-risk clusters were recorded in the southern hemisphere over a long period of time. This clustering is very specific and considerable, and may be attributed to synergistic interactions between environmental and geographic risk factors. Nonetheless, it was very difficult to evaluate the effect of these factors on the corresponding incidence and mortality clusters. In our literature review, we only found one study performed in Shandong province, China, which detected differences in the geographic distribution and risk clusters of PC mortality between urban and rural areas [14]. Generally, we hope that the results of this spatial analysis will provide a basis for investigators to explore the various spatial risk factors of incidence and mortality from PC in their future epidemiological studies.

The authors acknowledge the presence of several recognized limitations in this study that require emphasis. The primary limitation is that, despite employing methods to decrease bias in GBD 2019 estimates, such as correcting misclassifications and addressing incompleteness, the data quality in certain countries remains inadequate, leading to imprecise data. Consequently, additional studies are needed to investigate strategies for mitigating biases in the data. Secondly, it is important to note that the results of joinpoint regression analysis can be sensitive to parameter settings. Therefore, altering the parameters or analyzing additional data may result in changes to the observed patterns in disease burden trends. As an intriguing avenue for future investigations, it would be valuable to adjust the joinpoint regression parameters by selecting five different values within a plausible range for each parameter, and observe how these changes impact the interpretation of the data. Thirdly, because it is an ecological study based on aggregated data, the possible bias caused by ecological fallacy cannot be excluded. Thus, the obtained results should be interpreted with caution. Among the measures that can be taken to address such bias are ensuring homogeneity in the analysis groups and mitigating the impact of confounding factors when working with sociodemographic factors [49]. Lastly, our dataset lacks information on major risk factors associated with pancreatic cancer, such as smoking, diabetes, and obesity. Future analytical studies that incorporate spatial analysis to measure these factors may be essential in providing more conclusive evidence regarding their impact across different countries. Despite the aforementioned limitations, the use of geographical information and spatial analyses in modeling disease response can offer valuable insights for decision-makers to design tailored interventions.

## Conclusions

We presented a large, comprehensive, and up-to-date analysis of both incidence and mortality data related to PC across the globe, obtained from the GBD study 2019. This is the first explanatory study to identify high- and low-risk areas of PC outcomes over the past three decades. Our results elucidated significant spatial high- and low-risk clusters of incidence and mortality which can provide useful information to researchers and policymakers for a better understanding of the spatial epidemiology of PC outcomes by assessing the determinants of spatial distribution of this cancer. Overall, according to our analysis, the current worldwide unfavorable trends of PC since the early 1990s highlight an alarming scenario. Thereby, a prevention and control strategy for PC should contain recommendations for improving both the quality of life in patients and adopting healthier lifestyles. In addition to these efforts, persistent monitoring of outcomes, efficient allocation of resources for developing methods to early diagnosis of PC, reducing its modifiable risk factors, evaluating novel treatment strategies, and developing more effective therapies can help to tackle this lethal disease on a global scale.
